# CAR T cell therapy efficacy and safety in SLE: a systematic review and pooled analysis of 47 patients across 10 studies

**DOI:** 10.1007/s00210-025-04425-z

**Published:** 2025-08-14

**Authors:** Omima Ahmed Sayed, Mohamed Ellebedy, Mohamed Ahmed Abu-Alsaud, Amro Issam Bakri Meligy, Tamer A. Gheita

**Affiliations:** 1 Department of Rheumatology, Rehabilitation and Physical Medicine, Faculty of Medicine, Minia University, Minia, Egypt; 2 Faculty of Medicine, Sohag University, Sohag, Egypt; 3 Faculty of Medicine, Helwan University, Cairo, Egypt; 4 Faculty of Medicine, Kasr Al-Ainy, Cairo University, Cairo, Egypt; 5 Department of Rheumatology, Faculty of Medicine, Cairo University, Cairo, Egypt

**Keywords:** CAR T cell therapy, Systemic lupus erythematosus, Systematic review

## Abstract

Conventional therapies for systemic lupus erythematosus (SLE) often fail to achieve lasting remission. CAR T cell therapy, which depletes autoreactive B cells, offers a novel approach for refractory cases. We aim to consolidate the current evidence on CAR T cell therapy in the treatment of SLE. PubMed, Scopus, Web of Science, the Cochrane Library, and Clinical trials.gov were searched up to November 18, 2024, for SLE patients receiving CAR T cell therapy. Data on efficacy, safety, and treatment responses were analyzed. Ten studies involving 47 SLE patients were identified. Among those patients, 81% achieved lupus low disease activity state (LLDAS) with improvements in disease activity and serologic markers. B cell depletion was consistent, with immune recovery over time. Cytokine release syndrome (CRS) occurred in 87% but was mostly mild (grades 1–2). Immune effector cell-associated neurotoxicity syndrome (ICANS) occurred only in one patient and was mild (grade 1). CAR T cell therapy shows promise in refractory SLE, achieving durable remission with manageable toxicity. Further trials are needed to confirm long-term outcomes.

**Supplementary Information:**

The online version contains supplementary material available at 10.1007/s00210-025-04425-z.

## Introduction

Systemic lupus erythematosus (SLE) is a chronic autoimmune disease characterized by B cell dysfunction, leading to production of autoantibodies which play a pivotal role in the initiation and progression of disease. Managing SLE patients remains a therapeutic challenge due to its complex immunopathogenesis (Feng et al. 2020; Arnaud et al. 2024). Historically, SLE management relied on antimalarials, corticosteroids, and traditional immunosuppressants, which are effective in suppressing the ongoing inflammatory process but do not address the underlying immune dysfunction. The advent of biologic therapies has introduced more targeted approaches to treatment, but achieving sustained remission remains challenging (Ruiz-Irastorza and Bertsias 2020; Basta et al. 2020). Many SLE patients either experience refractory disease leading to marked morbidity or suffer from adverse effects associated with prolonged immunosuppression (Urowitz et al. 2012).

The emergence of chimeric antigen receptor (CAR) T cell therapy represents a paradigm shift in immunotherapy. Originally developed for hematologic malignancies, CAR T cell therapy involves the genetic modification of T cells to express receptors specific to antigens present on B cells. This approach targets and depletes pathogenic autoreactive B cells while sparing other components of the immune system, offering a potentially more targeted and durable therapeutic option compared to conventional B cell-depleting biologics. Given its ability to penetrate tissues more deeply, CAR T cell therapy has the theoretical advantage of achieving more comprehensive B cell depletion resulting in a “deep remission” state (Schett et al. 2023; Kretschmann et al. 2023; Lyu et al. 2024; Guffroy et al. 2024).

Preclinical models and early-phase clinical trials have demonstrated promising results with CAR T cell therapy in SLE, including significant clinical responses in patients with refractory disease (Mackensen et al. 2022; Jin et al. 2021). These findings suggest that CAR T cell therapy may represent an upgrade in the management of SLE, offering a promise to reset the immune dysregulation and as a result a drug-free remission. Despite encouraging preliminary findings, critical questions remain regarding the long-term safety, efficacy, and durability of response to CAR T cell therapy in SLE. This systematic review aims to consolidate current evidence on CAR T cell therapy in the context of SLE.

## Methodology

### Data sources and search strategy

Our systematic review was executed and reported in accordance with the Preferred Reporting Items for Systematic Reviews and Meta-Analyses (PRISMA) guidelines (Page et al. 2021). As this analysis was based on previously published studies, ethical approval and patient consent were not required. We conducted a systematic search of five electronic databases, PubMed, Scopus, Web of Science, the Cochrane Library, and Clinical trials.gov to identify relevant literature published up to November 18, 2024. Our search strategy employed a combination of keywords and medical subject headings (MeSH) combined with Boolean operators “AND” and “OR” to ensure comprehensive coverage of the literature. Specifically, we used the following search terms: (“CAR T cells” OR “CAR T-cells” OR “chimeric antigen receptor T Cell” OR “chimeric antigen receptor T-Cell” OR “chimeric antigen receptor T-lymphocyte” OR “chimeric T-Cell Receptor” OR “CAR-T cell therapy” OR “CAR-T therapy” OR “CAR-T”) AND (“systemic lupus erythematosus” OR SLE OR “lupus” OR “autoimmune disease” OR “autoimmune disorder”). The search strategy was adjusted to meet the requirements of each database. The results were imported into Rayyan software, a platform designed to facilitate the systematic review process, especially for screening and selecting studies.

### Study selection and eligibility criteria

The inclusion criteria were based on the Population, Intervention, Comparator and Outcome (PICO) framework (Schardt et al. 2007). Two independent investigators assessed all articles at the title and abstract level, after which the full text was read to confirm relevance. The conflicts were resolved by discussion. Only primary studies reporting SLE patients receiving CAR T cell therapy were included. There were no restrictions regarding geographical region or publication date of the included articles. The reference lists of retrieved studies were hand-searched to include additional eligible studies.

### Data extraction

Data from the included studies were extracted and recorded in a Microsoft Excel spreadsheet, which contained the following study information: study ID (first author’s last name and year of publication), study design, site, date of CAR T cell infusion, sample size, number of females and their percentage, age (in years), SLE duration (range; median), type of CAR T cells, dose, and follow-up duration. Additionally, we collected patient-by-patient data, including age (in years), sex, SLE duration (in years), organ involvement, type of CAR T cells, dose, follow-up duration (in days), previous medications including the prior use of anti B cell therapies, CAR T cell expansion, and the day of its peak, SLEDAI-2K (baseline and at the end of follow-up), physician global assessment (PGA), ability to discontinue all SLE-related medications, and achievement of LLDAS (lupus low disease activity state) or DORIS (Definitions of Remission in SLE), anti-dsDNA, and complement levels, cytokine release syndrome (CRS) and its grade, management of CRS, and immune effector cell-associated neurotoxicity syndrome (ICANS). For ongoing trials, we extracted the following information for each trial: ClinicalTrials.gov ID, phase, start date, intervention, target number of participants, and trial status.

### Statistical analysis

Since this review is primarily descriptive, we utilized descriptive statistics, reporting means or medians for continuous variables and frequencies with percentages for categorical variables.

### Risk of bias assessment

The quality of included case reports was assessed using the Joanna Briggs Institute (JBI) Critical Appraisal Checklist for case reports (Munn et al. 2020), covering patient demographics, clinical history, presentation, diagnostics, interventions, post-intervention outcomes, adverse events, and takeaway lessons. Wang et al. and Muller et al. were assessed using the JBI Critical Appraisal Checklist for case series (15), evaluating inclusion criteria, measurement reliability, identification methods, participant inclusion, demographic and clinical data reporting, outcome reporting, site demographic reporting, and statistical analysis. Risk of bias (ROB) was independently evaluated by two investigators, with discrepancies resolved through discussion. Conference abstracts were excluded from quality assessment.

## Results

### Search results

**Fig. 1:**
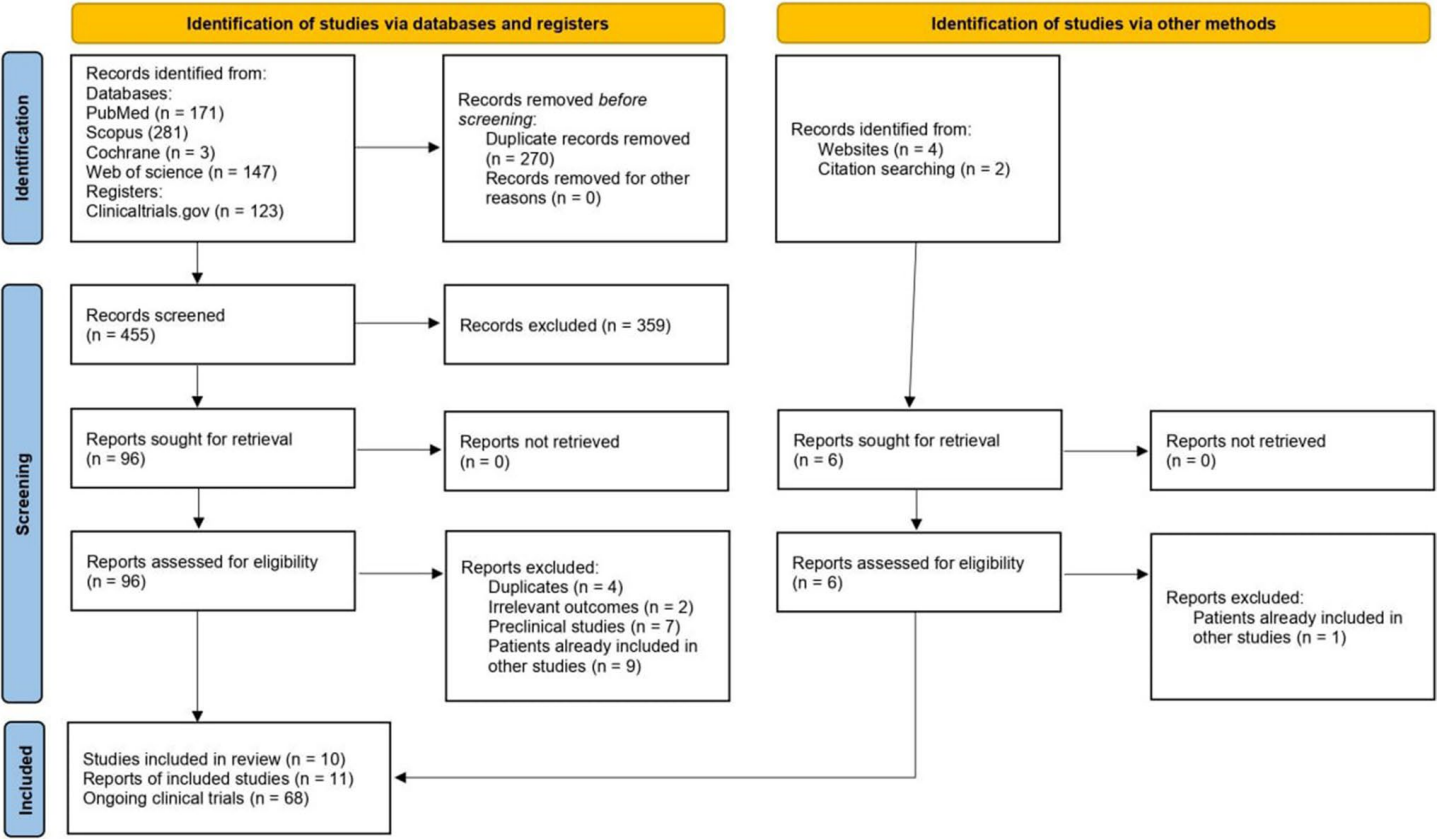
2020 PRISMA flow diagram of the study selection

Our search strategy identified 725 studies from PubMed (*n* = 171), Scopus (*n* = 281), Web of Science (*n* = 147), Cochrane Library (*n* = 3), and Clinical trials.gov (*n* = 123). A total of 270 duplicate articles were removed, followed by the exclusion of 359 studies during the title and abstract screening. After full-text screening, ten studies (11 reports) and 68 clinical trials were included in this systematic review (Fig. 1).

### Study characteristics

The eleven included reports (Marasco et al. 2024; Podoll et al. 2024; Hagen et al. 2024; Li et al. 2024; Krickau et al. 2024; He et al. 2025; Müller et al. 2024; Feng et al. 2023; Cortés-Hernández et al. 2023, 2024; Wang et al. 2024) comprised single-arm clinical trials (*n* = 4), case series (*n* = 1), and case reports (*n* = 6). Three of the clinical trials were presented as conference abstracts, with two of them (Cortés-Hernández et al. 2023, 2024) being different reports of the same trial. Additionally, two of the case reports were conference abstracts (Table 1).

**Table 1:**
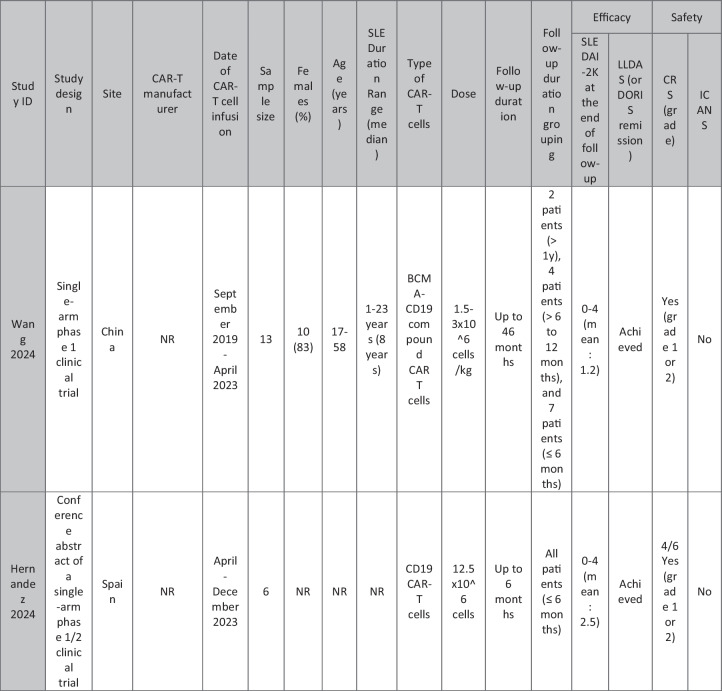

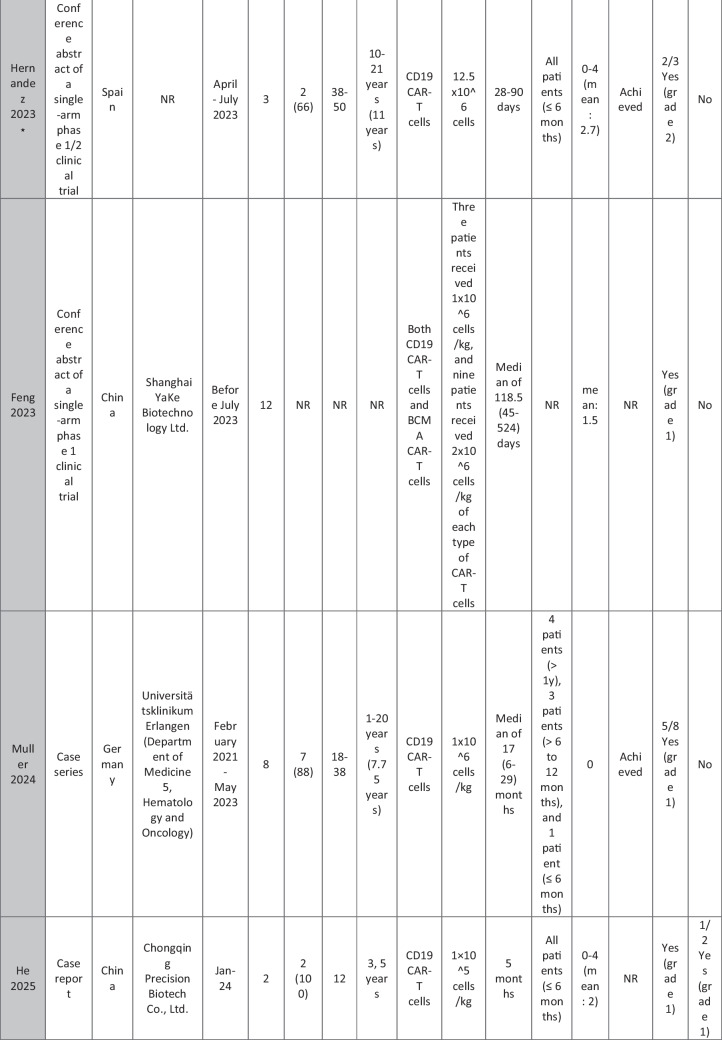

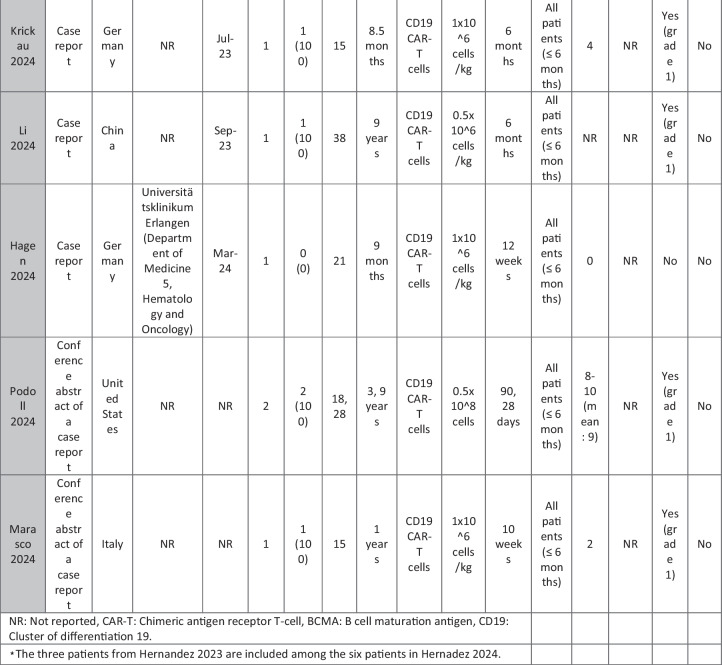
Summary of the included studies

### Patient baseline characteristics

A total of 47 patients with SLE were included from 10 studies conducted across China, Spain, Germany, Italy, and the USA. Patient age ranged from 12 to 58 years (median 29 years, reported in 8 out of 10 studies), with four studies including pediatric patients (*n* = 6). 78% were females (24/31 patients); three studies did not report sex distribution. The median disease duration was 6.5 years, with a range of 0.71 to 23 years (32/47 patients). All patients had received conventional immunosuppressive therapy, including glucocorticoids (100%), hydroxychloroquine (85%), mycophenolate (70%), cyclophosphamide (65%), and biologic agents such as rituximab and belimumab. Two patients (Wang et al.) had comorbid refractory diffuse large B cell lymphoma. Baseline SLEDAI-2K scores ranged from 4 to 23, with a median of 12 (IQR 8.5–16) (data available from all studies except Feng and Müller). Lupus nephritis was the most common disease manifestation, reported in 28 patients. Other manifestations included hematological (10 patients), pulmonary (6 patients), cardiac (5 patients), and neurological (1 patient) involvement. Most patients had multi-system involvement and had failed at least two lines of therapy prior to enrollment (Table 2).

**Table 2:**
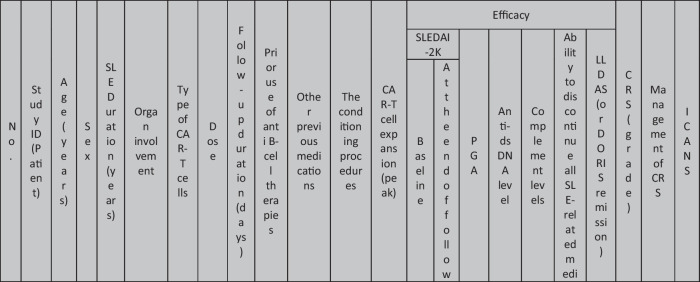

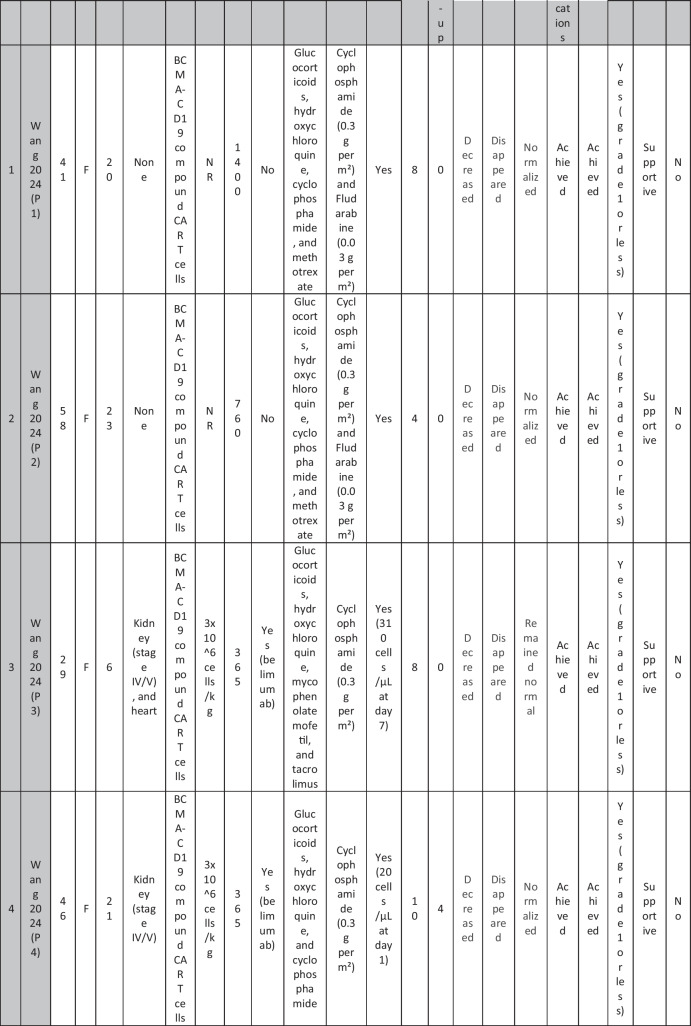

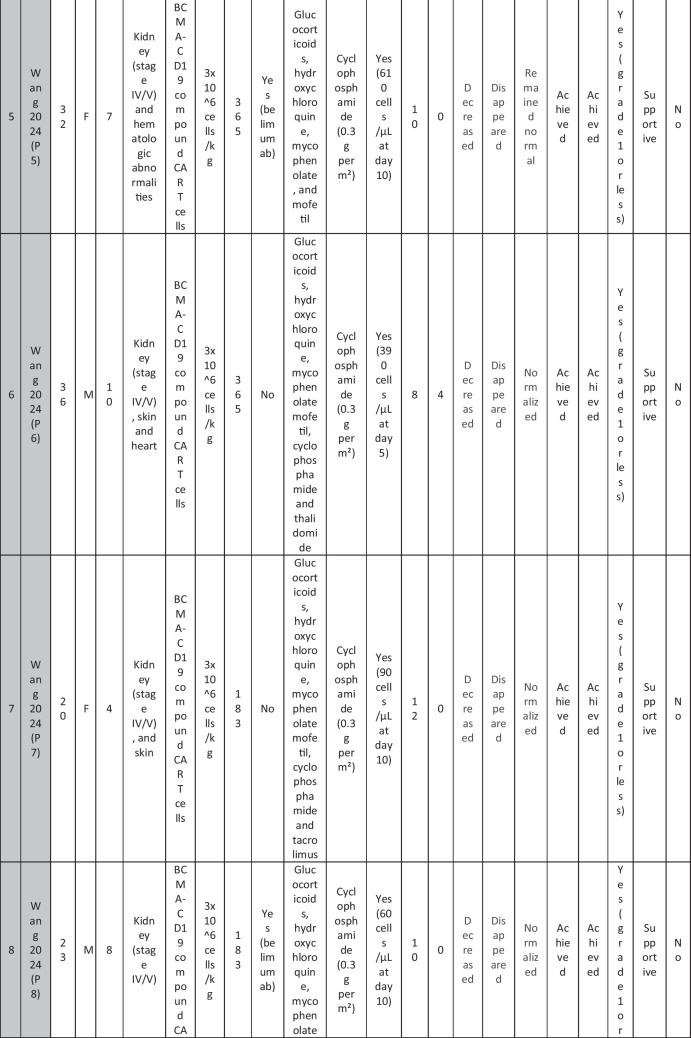

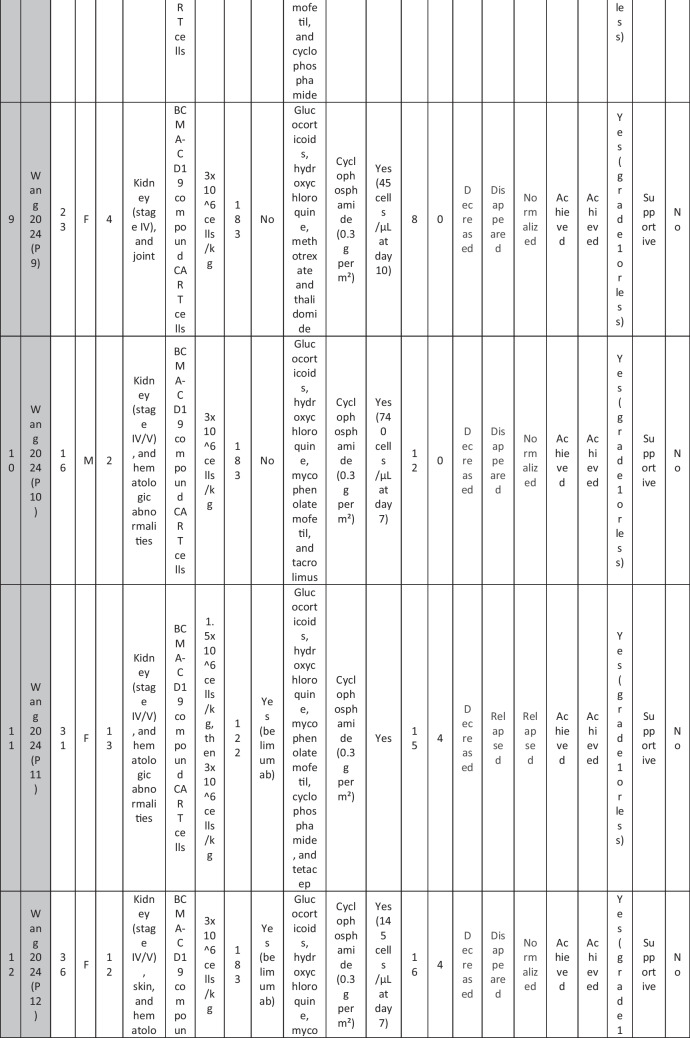

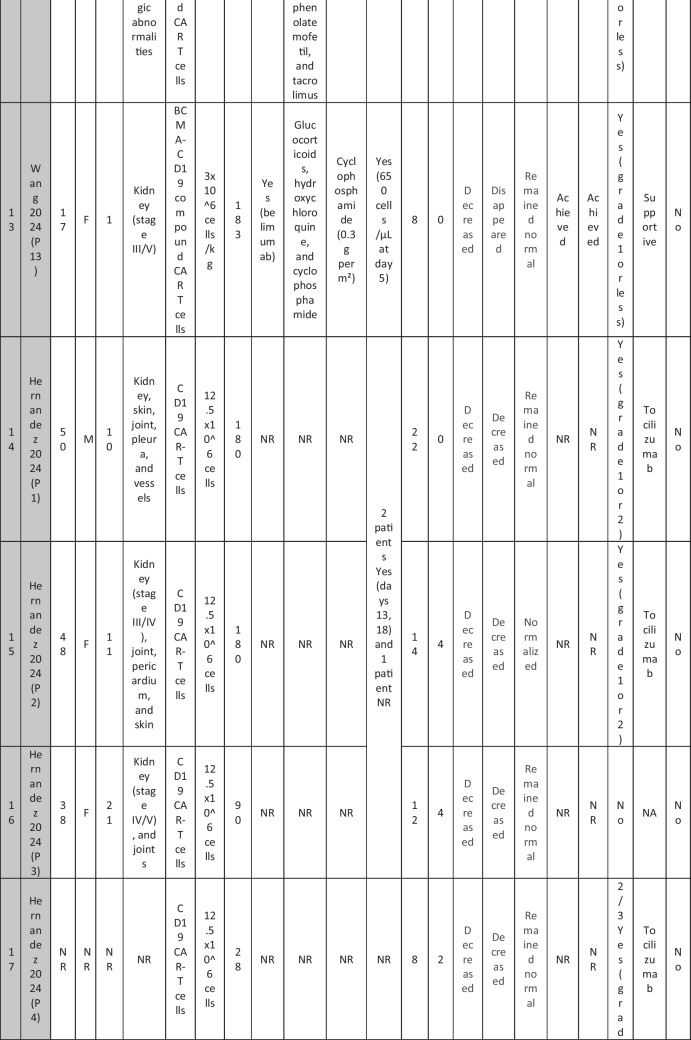

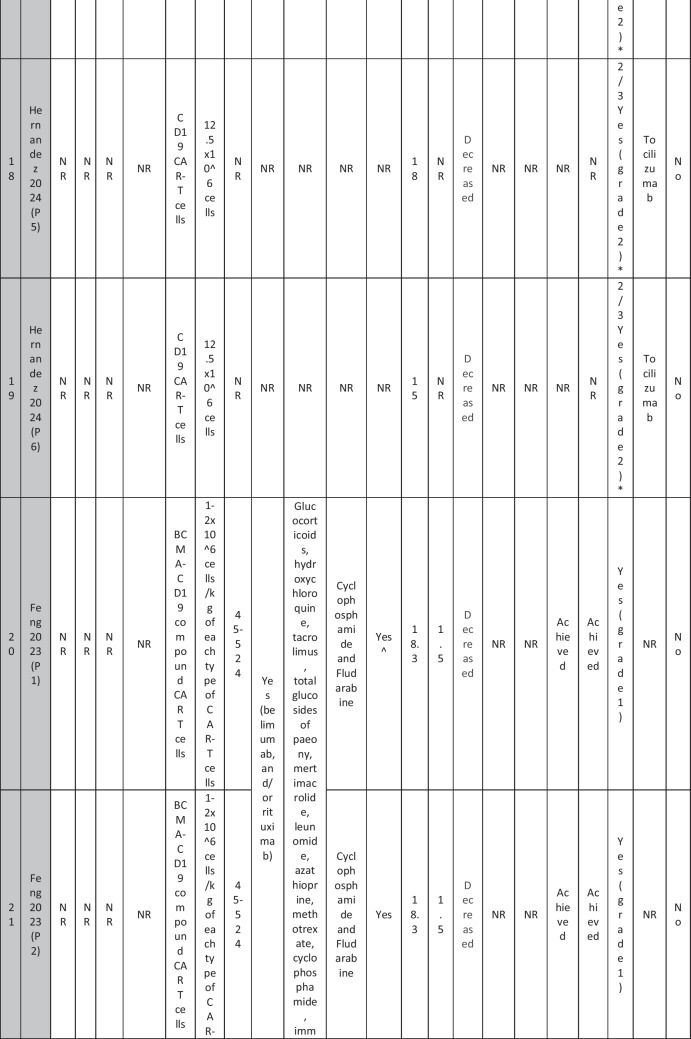

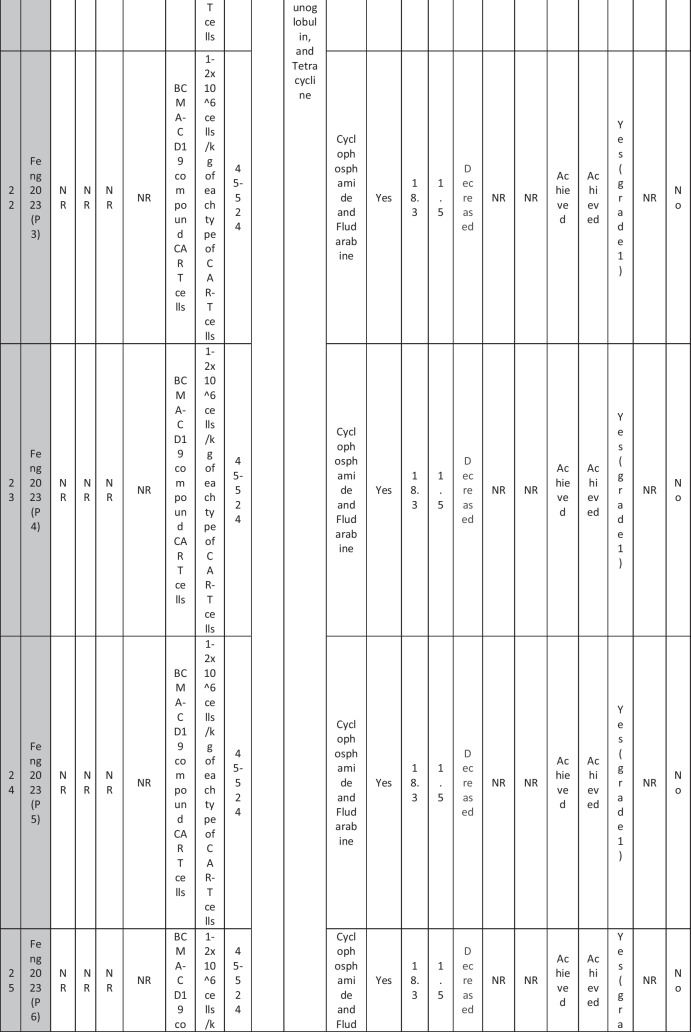

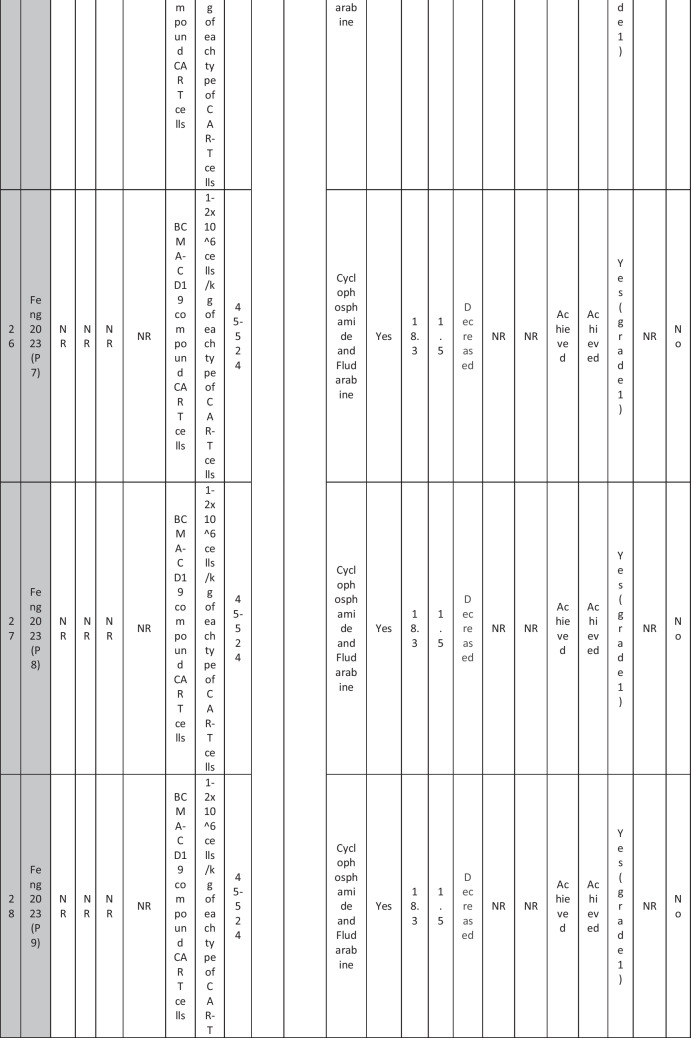

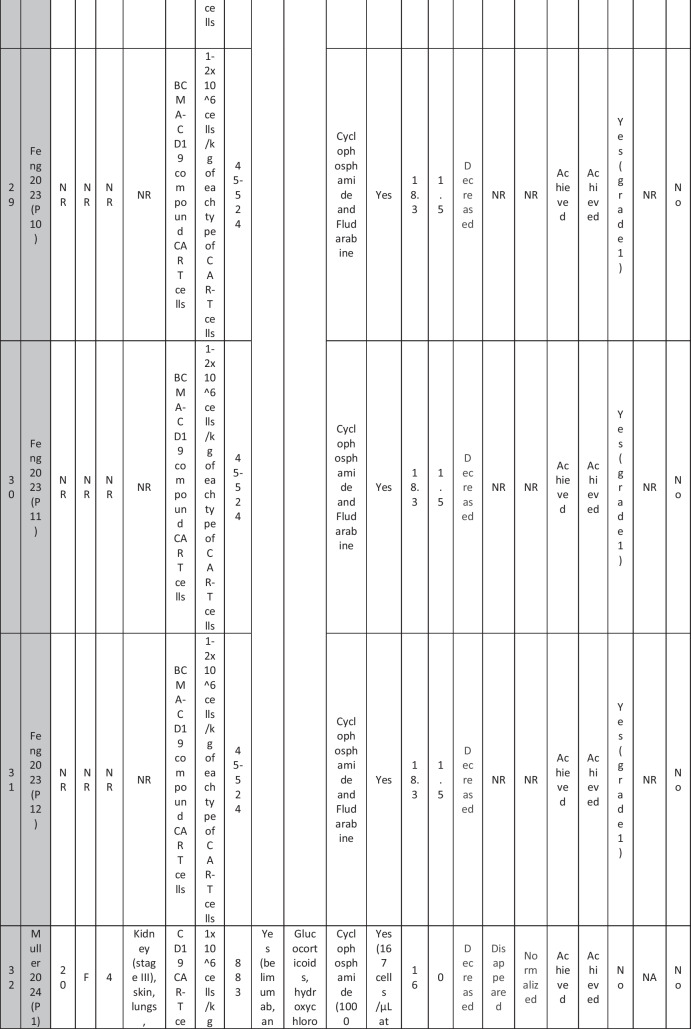

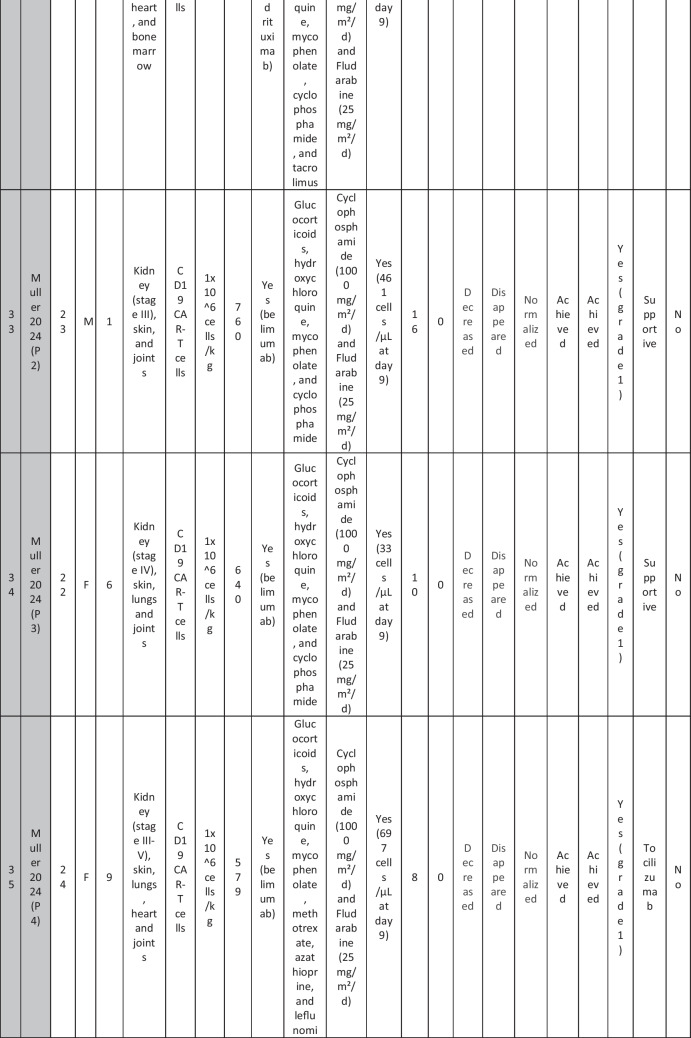

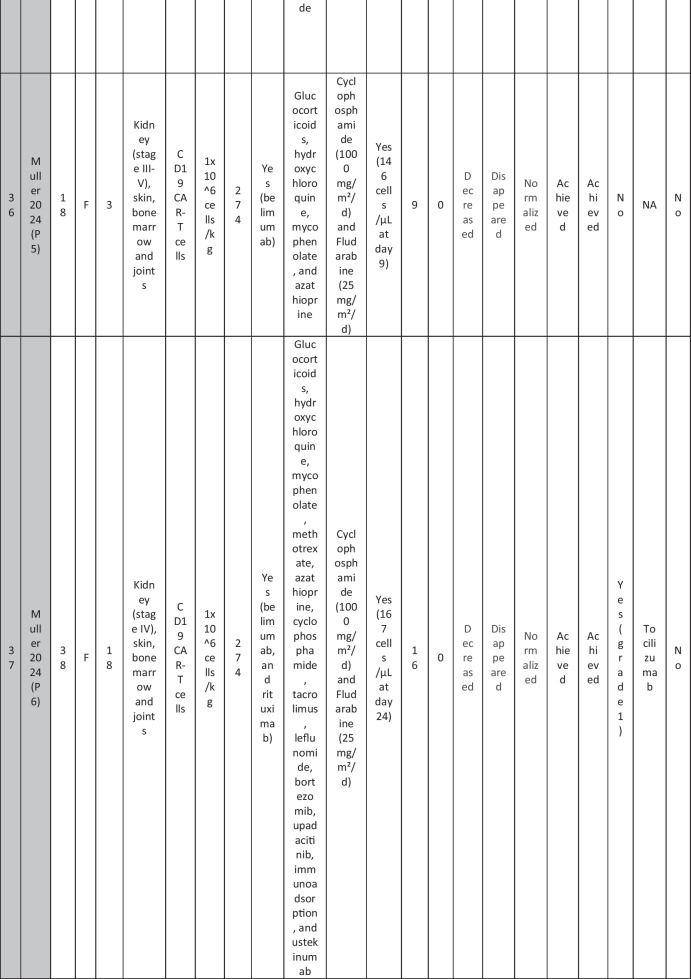

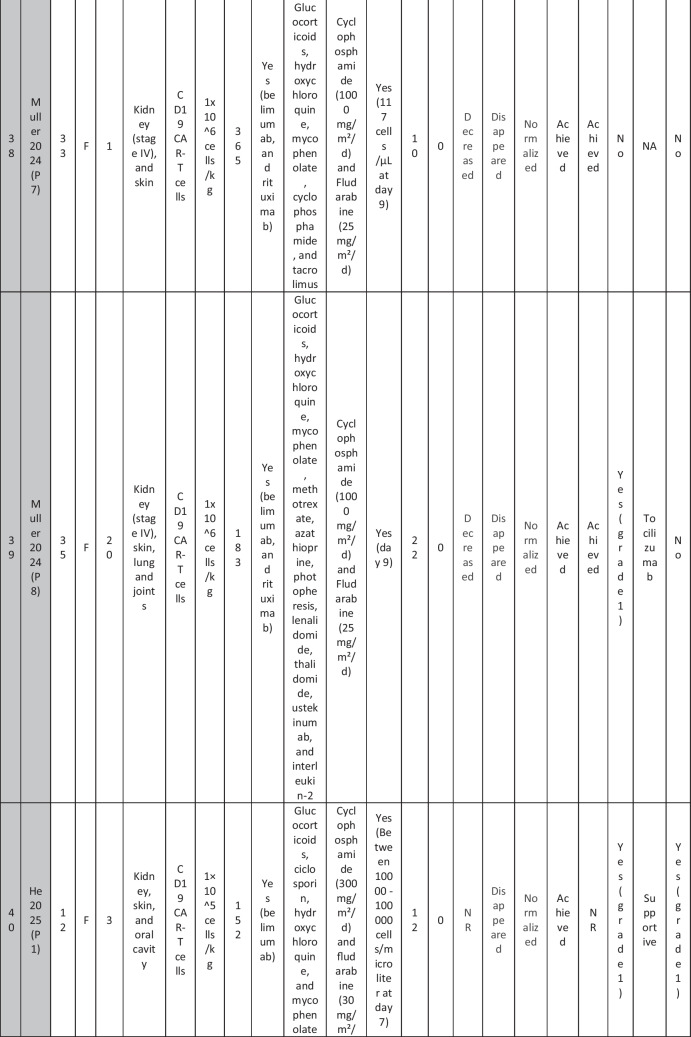

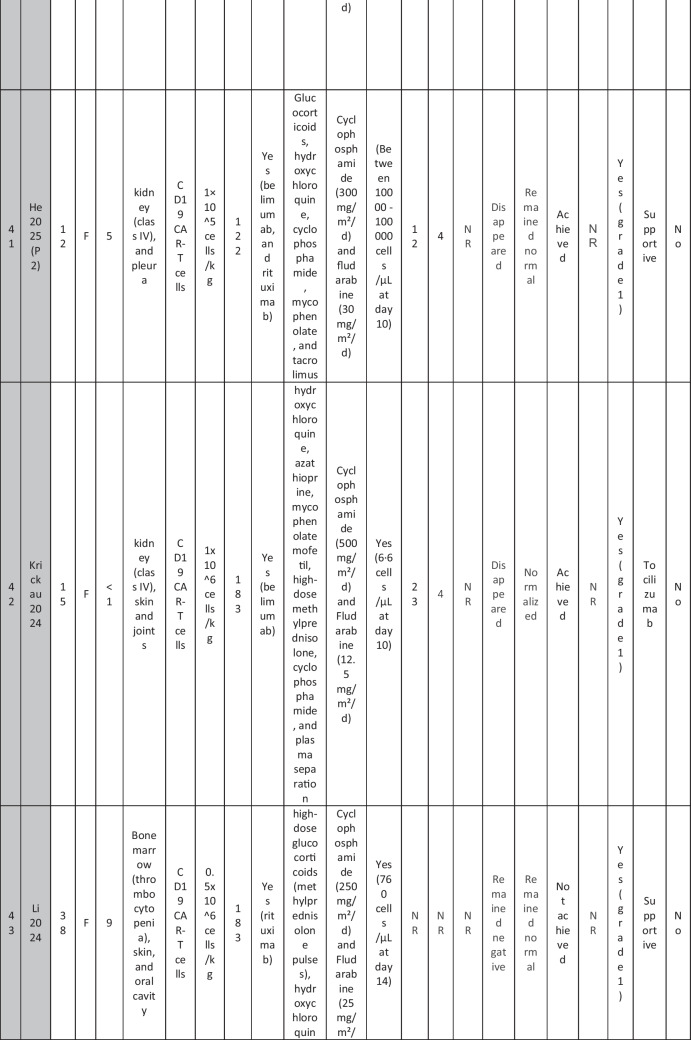

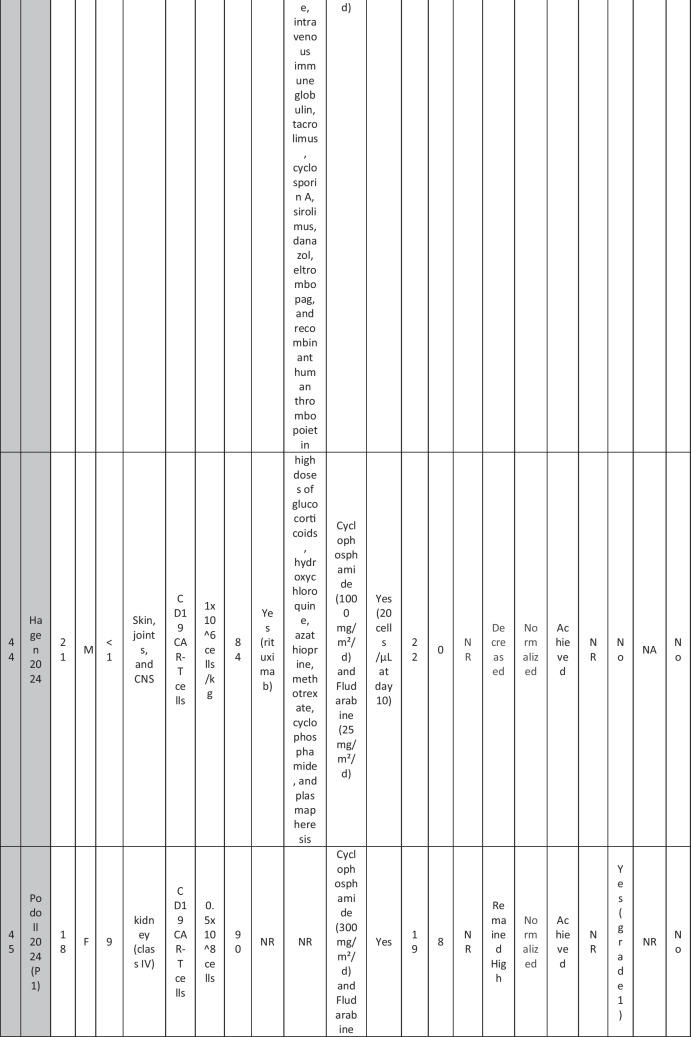

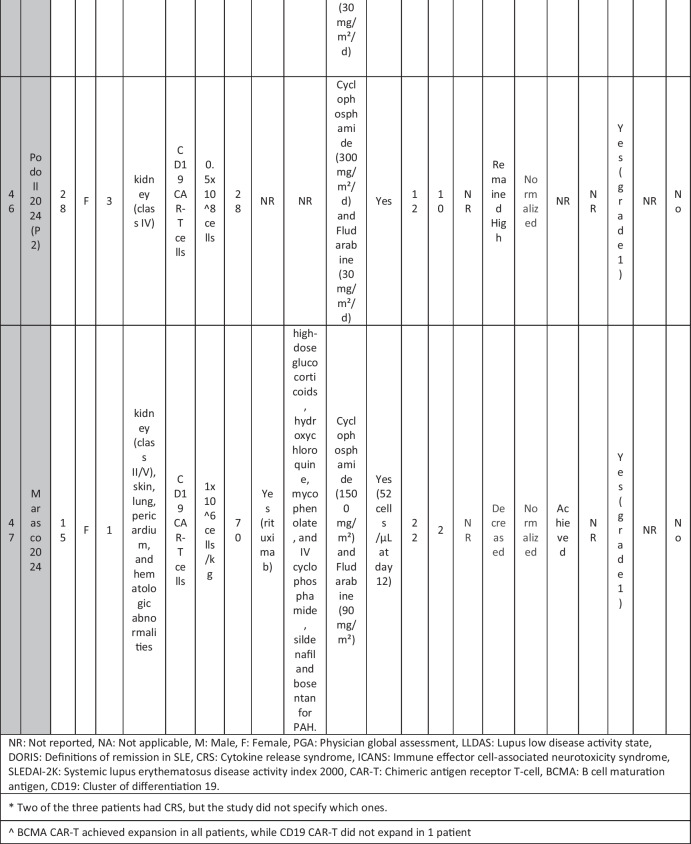
Patient-by-patient data

### CAR T cell therapy dosing regimens

A total of eight studies investigated CD19-targeted CAR T cell therapy, with varying dosing regimens. In four studies, CD19 CAR T cells were administered at a dose of 1 × 10⁶ cells/kg. Two studies used lower doses, ranging from 0.5–1 × 10^5^ cells/kg, while two other studies employed fixed-dose regimens of 12.5–100 × 10⁶ cells per infusion. In addition, two trials (25) (23) (*n* = 25 patients) explored BCMA-CD19 compound CAR T cell therapy with different dosing approaches. Wang et al. administered 3 × 10⁶ cells/kg to all patients except one, who received a reduced initial dose of 1.5 × 10⁶ cells/kg due to severe lymphopenia. This patient later experienced relapse and was retreated with a second infusion of 3 × 10⁶ cells/kg. Feng et al. used 1 × 10⁶ cells/kg in three patients and 2 × 10⁶ cells/kg in nine patients. Regarding the conditioning regimens, 30 patients received cyclophosphamide and fludarabine, 11 received cyclophosphamide alone, and for 6 patients, the regimen was not reported.

### Efficacy of CAR T cell therapy

Follow-up durations ranged from 28 days to 46 months. Thirty-eight SLE patients met the LLDAS/DORIS criteria for remission. At the time of their last follow-up, most patients were able to discontinue all SLE-related medications and maintained drug-free remission. SLEDAI-2K scores among these patients ranged from 0 to 10, with a median of zero. A 31-year-old female reported by Wang et al. [p11] experienced severe bone marrow suppression. Following an initial low dose of BCMA-CD19 compound CAR T cells, relapse was observed, necessitating a second full-dose administration. However, recurrence of SLE was observed following an increase in autoantibodies and a decline in complement levels, requiring the resumption of SLE treatment. Additionally, in case reports of two patients with grade IV lupus nephritis, both maintained SLEDAI scores > 4 (P1: 8 at day 90; P2: 10 at day 28) along with elevated anti-dsDNA levels (Podoll et al.).

Refractory severe lupus nephritis (grades III and IV) was the most prevalent manifestation among SLE patients receiving CAR T cell therapy. Most studies reported reductions in proteinuria and normalization of renal function. However, a few cases reported rebound proteinuria after initial improvement, such as P4 in Müller et al., whose renal biopsy revealed podocytopathy, and P6 in Wang et al., whose 24-h proteinuria at last follow-up was 8 g, compared to a baseline of 2.8 g/24 h. Low-level proteinuria, reflecting irreversible renal damage, was observed in studies by He et al., Feng et al., and Krickau et al.

Wang et al. reported two patients with diffuse large B cell lymphoma (DLBCL) and SLE. Both achieved undetectable autoantibody titers, symptom-free and drug-free remission, and demonstrated B cell recovery within normal ranges.

Refractory immune thrombocytopenia (ITP) secondary to SLE showed promising results (Li, Wang p3). Hagen et al. treated a case of transverse myelitis and neuropsychiatric lupus with CAR T therapy. Despite prior treatment with rituximab, cyclophosphamide, and plasmapheresis, the patient developed progressive weakness leading to spastic paraplegia. Following CAR T therapy, the patient regained the ability to walk with support by week 10, with MRI showing improvement in brain and spinal cord lesions. CAR T cells successfully crossed the blood–brain barrier and eliminated B cells in the cerebrospinal fluid. Serologic improvement was observed in all patients. Anti-dsDNA titters declined in eight patients (1 Wang, 2 Podoll, 1 Hagen, 1 Marasco, 3 Hernández) and became negative in 23 patients (data not reported for Feng and three Hernández cases), correlating with B cell aplasia. Complement levels (C3/C4) normalized in 31 patients (data not reported for Feng and the second patient in He).

### Adverse effects of CAR T cell therapy

CRS was reported in 41 patients, all experiencing grades 1–2 CRS, which resolved with supportive therapy. Nine patients required tocilizumab. ICANS was reported in only one patient on day 8 post-CAR T therapy, presenting with tremors, dysgraphia, mild speech difficulty, and lethargy; symptoms resolved following steroid therapy (P1 He). A few patients developed infections, predominantly upper respiratory tract infections, all resolving with supportive treatment; no patients required intensive care unit (ICU) admission. Transient neutropenia, anemia, and thrombocytopenia were common but self-limiting.

### Ongoing clinical trials

A total of sixty-eight trials are actively recruiting, with planned target sample sizes ranging from 12 to 75 participants. CAR T cell therapies targeting CD19 (*n* = 15), BCMA (*n* = 5), BAFF-R (*n* = 3), and dual antigens (*n* = 3) are under investigation. Innovative approaches include CAR-NK cells (*n* = 2), CRISPR-edited CAR T cells (*n* = 1), and autoantigen-specific BCR-targeted CAR T cells (*n* = 1) (Table S1).

### Risk of bias assessment

All included case reports met the JBI criteria, indicating a low risk of bias. However, Wang et al. did not include participants consecutively, while Muller et al. had incomplete participant inclusion. Despite these limitations, both studies demonstrated robust measurement reliability, valid condition identification methods, and comprehensive outcome reporting (Tables S2 and S3).

## Discussion

Our systematic review and pooled analysis of 47 SLE patients treated with CAR T cell therapy demonstrate transformative potential, with most patients achieving remission or low disease activity state along with serologic normalization. This positions CAR T cell therapy as a breakthrough for refractory SLE, particularly in severe phenotypes like lupus nephritis. However, several concerns regarding the heterogeneity in responses, variability in relapse rates, and safety risks remain. While further understanding of what happens after the reconstitution of B cell tolerance is needed, CAR T cell therapy presents a revolutionary step in the management of SLE. SLE is characterized by the presence of multiple autoantibodies against nuclear components and systemic inflammation, which lead to the damage of multiple organs. Abnormal maturation and activation of B cells play a pivotal role in the immunopathogenesis of SLE in both antibody-dependent and antibody-independent manners (Canny and Jackson 2021). Current SLE treatments, including corticosteroids, conventional immunosuppressants, and biologics (e.g., rituximab and belimumab), suppress immune hyperactivity but fail to eliminate autoreactive B cell clones driving pathogenesis (Basta et al. 2020; Katarzyna et al. 2023).

CAR T cell therapy uniquely enables antigen-selective immune reset by eradicating CD19 + B cells, plasmablasts, and memory B cells, unlike rituximab, which spares CD19-negative plasma cells, thus offering a more targeted approach by selectively depleting auto-reactive B cells. Unlike monoclonal therapy, CART cells can induce longer-lasting immune modulation, potentially leading to sustained remission (Stockfelt et al. 2025; Rampotas et al. 2025; Zhou et al. 2024). This explains the sustained anti-dsDNA normalization and complement recovery in most of our pooled SLE patients, suggesting restored immune tolerance rather than transient immunosuppression. Despite variation in dosing regimens and follow-up durations, consistent improvements in SLEDAI-2K scores and proteinuria reduction are noted (Mougiakakos et al. 2024). The therapy’s ability to penetrate sanctuary sites (e.g., renal tubulointerstitium and CSF) also underpins its success in neuropsychiatric lupus cases, as seen in Hagen et al.’s patient with transverse myelitis (Hagen et al. 2024). A key strength of CAR T cell therapy is its capacity to induce durable remission in refractory cases, with most patients discontinuing all immunosuppressants. However, relapses occurred, often linked to incomplete B cell depletion or CD19-negative plasma cell persistence. For example, Müller et al. reported residual proteinuria in a patient with podocytopathy despite B cell aplasia, implicating non-B cell mediators in end-organ damage (Müller et al. 2024). Dual-target CAR T cells (e.g., BCMA-CD19) showed promise in Wang et al.’s cohort, where both SLE and lymphoma patients achieved remission, suggesting broader antigen targeting may reduce relapse risk. Retreatment was required in one patient after B cell repopulation, highlighting the need for strategies to address persistent plasma cell reservoirs, such as adjunctive proteasome inhibitors (Wang et al. 2024).

CRS is a potentially serious side effect that typically occurs within 1–14 days after CAR T cell infusion, as the engineered immune cells expand and attack targeted cells. This condition arises from an excessive immune response, where interferon-γ is released, prompting macrophages to produce inflammatory mediators like interleukin-6 (IL-6), tumor necrosis factor-α (TNF-α), and IL-10. The IL-6/soluble IL-6 receptor complex activates cells via gp130, while additional cytokines such as IL-1, IL-5, IL-8, and GM-CSF further intensify the inflammatory cascade. This surge in cytokines can lead to symptoms like fever, low blood pressure, low oxygen levels, and flu-like symptoms. In severe cases, CRS can cause organ failure and may be life-threatening. Treatment often involves tocilizumab, corticosteroids, and supportive care to manage symptoms and prevent complications (Morris et al. 2022). Cytokine release syndrome occurred in 87% of patients, albeit low-grade (1–2), contrasting sharply with oncology cohorts where severe CRS (grade ≥ 3) occurs in 20–50% (Sheth and Gauthier 2021; Chen et al. 2023; Zhang et al. 2022). This divergence may reflect lower inflammatory burden in SLE or differences in T cell activation thresholds. ICANS was rare, and no ICU admissions occurred, supporting feasibility in autoimmunity.

However, prolonged B cell aplasia led to severe infections, including pneumonia and cytomegalovirus reactivation. Standardized prophylaxis (e.g., IVIG and antimicrobials) and vigilant monitoring during immune reconstitution are critical, especially in pediatric patients, where long-term impacts on vaccination responses remain unknown. Vaccination should be started after resolution of B cell aplasia, taking into consideration the IVIG replacement therapy that reduces the immunogenicity of vaccines (Los-Arcos et al. 2021). Beyond these immediate adverse events, several drawbacks and limitations must be acknowledged. The durability of response and relapse risk, While CAR-T therapy induces remissions lasting up to 46 months, longer follow-up is needed to assess late relapses and durability of response. B cell repopulation may reintroduce autoimmunity if the initial triggers re-break the tolerance (Guffroy et al. 2024). Also, by being highly expensive, CAR T cell therapy is inaccessible in resource-limited settings. CAR T cell therapy involves a time-consuming process of T cell collection, modification, expansion, and reinfusion, limiting its accessibility (Ohno and Nakamura 2024).

This systematic review has several limitations. It included only SLE patients from case reports, case series, and non-randomized phase 1 trials. Due to heterogeneity in baseline and follow-up data and inconsistent outcomes reporting, meta-analysis and effect size estimation were not feasible. The single-arm design, small sample sizes, lack of control groups, and short follow-up contribute to a high risk of bias, including selection and publication bias. These limitations underscore the need for randomized controlled trials with standardized methodologies to assess the long-term efficacy and safety of CAR T cell therapy.

## Conclusion

CAR T cell therapy represents a paradigm shift in SLE management, offering deep, drug-free remission in patients with refractory disease. While challenges in relapse, safety, and accessibility persist, its mechanistic superiority over conventional therapies underscores its potential to redefine autoimmune treatment. Strategic integration of biomarker-guided protocols, cost-reduction technologies, and global registries will be pivotal to democratize access and solidify CAR T cell therapy as a cornerstone of SLE care.

## Supplementary Information

Below is the link to the electronic supplementary material.

Supplementary file1 (DOCX 30 KB)

Supplementary file2 (DOCX 15 KB)

Supplementary file3 (DOCX 15 KB)

## Data Availability

All source data for this work (or generated in this study) are available upon reasonable request.
