# Supplementary material for: CAR T cell therapy efficacy and safety in SLE: a systematic review and pooled analysis of 47 patients across 10 studies
Source: Naunyn Schmiedebergs Arch Pharmacol. 2025 Aug 14;399(2):1565–88. doi: 10.1007/s00210-025-04425-z (PMC12901168; doi:10.1007/s00210-025-04425-z)
Supplement: Supplementary file 1 — Supplementary file1 (DOCX 30 KB) [file 210_2025_4425_MOESM1_ESM.docx]

| ClinicalTrials.gov ID | Phase | Starting date | Intervention | Target number of participants | Status |
| --- | --- | --- | --- | --- | --- |
| NCT03030976 | Phase 1 | Mar-17 | Cyclophosphamide - anti-CD19-CAR-T cells | 5 | Unknown |
| NCT05030779 | Phase 1 | Oct-21 | CD19/BCMA CAR T-cells | 9 | Unknown |
| NCT05085418 | Phase 1 | Nov-21 | CD19/BCMA CAR T-cells | 9 | Unknown |
| NCT06316791 | Phase 1 | Dec-21 | single dose of CNCT19 | 24 | Recruiting |
| NCT05474885 | Phase 1 | Apr-22 | BCMA-CD19 CAR T cells | 15 | Recruiting |
| NCT05459870 | Phase 1 & 2 | Jul-22 | 4SCAR T cells | 30 | Recruiting |
| NCT06342960 | Phase 1 & 2 | Dec-22 | KYV-101 anti-CD19 CAR-T cell therapy - Standard lymphodepletion regimen | 32 | Recruiting |
| NCT05765006 | Phase 1 | Feb-23 | Relma-cel | 24 | Recruiting |
| NCT05798117 | Phase 1 & 2 | Feb-23 | YTB323 | 24 | Recruiting |
| NCT05858684 | Phase 1 | May-23 | GC012F injection | 18 | Recruiting |
| NCT05846347 | Phase 1 | May-23 | GC012F injection | 15 | Recruiting |
| NCT05859997 | NA | May-23 | BRL-301 | 15 | Enrolling by invitation |
| NCT06347718 | Phase 1 & 2 | Jul-23 | anti-CD19 CAR T cell | 24 | Recruiting |
| NCT05988216 | NA | Aug-23 | BRL-301 | 12 | Recruiting |
| NCT06010472 | Phase 1 | Aug-23 | Anti-CD19 CAR NK cells (KN5501) | 12 | Recruiting |
| NCT06056921 | Phase 1 | Aug-23 | CD19 targeted CAR-T cells | 24 | Recruiting |
| NCT05869955 | Phase 1 | Sep-23 | CC-97540 - Fludarabine - Cyclophosphamide - Tocilizumab | 129 | Recruiting |
| NCT06150651 | Phase 1 | Dec-23 | CAR T-cell therapy | 6 | Recruiting |
| NCT06222853 | Phase 1 | Jan-24 | anti-CD19-CAR-T cells | 18 | Recruiting |
| NCT06333483 | Phase 1 | Feb-24 | Obecabtagene autoleucel (obe-cel) | 12 | Recruiting |
| NCT06277427 | NA | Feb-24 | PRG-1801 (AR-T against BCMA） | 24 | Recruiting |
| NCT06038474 | Phase 2 | Feb-24 | Descartes-08 | 30 | Recruiting |
| NCT06153095 | Phase 1 & 2 | Feb-24 | IMPT-514 | 30 | Recruiting |
| NCT06121297 | Phase 1 & 2 | Feb-24 | CABA-201 | 12 | Recruiting |
| NCT06297408 | Phase 1 | Mar-24 | Relma-cel | 24 | Not yet recruiting |
| NCT06285279 | Phase 1 | Mar-24 | FKC288 | 24 | Recruiting |
| NCT06310811 | NA | Mar-24 | RD06-04 Cells injection | 12 | Recruiting |
| NCT06318533 | Phase 1 | Mar-24 | anti-CD19 CAR NK cells | 15 | Recruiting |
| NCT06249438 | Phase 1 | Mar-24 | CD20/BCMA-directed CAR-T cells | 30 | Recruiting |
| NCT06361745 | NA | Apr-24 | T cell injection targeting CD19 chimeric antigen receptor | 10 | Recruiting |
| NCT06503224 | NA | Apr-24 | Anti-BCMA and CD19 CART | 18 | Recruiting |
| NCT06138132 | Phase 1 | Apr-24 | KYV-101 anti-CD19 CAR-T cell and Standard lymphodepletion regimen | 12 | Recruiting |
| NCT06279923 | Phase 1 | Apr-24 | CD19-BAFF Targeted CAR T-cells | 45 | Recruiting |
| NCT06373081 | NA | Apr-24 | Anti-CD19-CD3E-CAR-T cells | 6 | Recruiting |
| NCT06294236 | Phase 1 | Apr-24 | SC291 | 36 | Recruiting |
| NCT06417398 | Phase 1 | May-24 | T cell injection targeting CD19 chimeric antigen receptor | 10 | Not yet recruiting |
| NCT06420154 | Phase 1 | May-24 | anti-CD19-CAR-T cells | 9 | Not yet recruiting |
| NCT06428188 | Phase 1 & 2 | May-24 | BCMA/CD19 CAR-T cells | 60 | Recruiting |
| NCT06462144 | Phase 1 | Jun-24 | IMPT-514 CART Cell Injection | 36 | Recruiting |
| NCT06557265 | Phase 1 | Jun-24 | NKX019 - Cyclophosphamide | 21 | Recruiting |
| NCT06106906 | Phase 1 & 2 | Jun-24 | CD19 CAR-T cell infusion | 15 | Recruiting |
| NCT06497387 | Phase 1 | Jul-24 | PRG-1801 | 30 | Recruiting |
| NCT06518668 | Phase 1 | Jul-24 | NKX019 - Cyclophosphamide LD | 6 | Recruiting |
| NCT06350110 | Phase 1 & 2 | Jul-24 | CD19- BCMA CAR-T cells | 75 | Recruiting |
| NCT06373991 | Phase 1 | Jul-24 | ATHENA CAR-T - Fludarabine - Cyclophosphamide | 12 | Not yet recruiting |
| NCT06513429 | NA | Jul-24 | IM19 CAR-T cells | 3 | Recruiting |
| NCT06152172 | Phase 1 | Aug-24 | KYV-101 - Cyclophosphamide - Fludarabine | 24 | Recruiting |
| NCT06189157 | Phase 1 & 2 | Aug-24 | MB-CART19.1 | 29 | Recruiting |
| NCT06530849 | Phase 1 & 2 | Aug-24 | GC012F Injection | 118 | Recruiting |
| NCT06548620 | Phase 1 | Aug-24 | RD06-04 cell infusion | 18 | Not yet recruiting |
| NCT06567080 | Phase 1 | Sep-24 | JWCAR201 | 15 | Not yet recruiting |
| NCT06581198 | Phase 2 | Sep-24 | Rapcabtagene autoleucel Regimen 1 | 144 | Recruiting |
| NCT06549296 | Phase 1 | Sep-24 | RD06-04 CART Cell Injection | 12 | Recruiting |
| NCT06548607 | Phase 1 | Sep-24 | RD06-04 CART Cell Injection | 20 | Recruiting |
| NCT06585514 | Phase 1 & 2 | Oct-24 | CD19 CAR-T cells | 18 | Recruiting |
| NCT06653556 | Phase 1 | Oct-24 | LCAR-AIO T cells | 34 | Not yet recruiting |
| NCT06465147 | Phase 1 | Oct-24 | SCRI-CAR19v3 | 12 | Recruiting |
| NCT06691152 | Phase 1 | Oct-24 | CD19 Universal CAR-T cells | 18 | Recruiting |
| NCT06429800 | Phase 1 | Nov-24 | ATA3219 | 52 | Not yet recruiting |
| NCT06375993 | Phase 1 | Nov-24 | ADI-001 - Fludarabine - Cyclophosphamide | 180 | Recruiting |
| NCT06661811 | NA | Nov-24 | CAR-T | 12 | Not yet recruiting |
| NCT06680388 | Phase 1 | Nov-24 | CD19 CAR-T | 15 | Not yet recruiting |
| NCT06688799 | Phase 1 & 2 | Nov-24 | CD19 CAR-T cells | 18 | Not yet recruiting |
| NCT06681337 | Phase | Nov-24 | BCMA CART + CD19 CART | 10 | Not yet recruiting |
| NCT06685042 | Phase 1 & 2 | Dec-24 | CAR T cell | 8 | Not yet recruiting |
| NCT06340750 | Phase 1 | Jan-25 | LMY-920 | 18 | Not yet recruiting |
| NCT06544330 | Phase 1 | Mar-25 | SYNCAR-001 - STK-009 | 42 | Recruiting |
| NCT06377228 | Phase 1 | May-25 | TAK-007 and Chemotherapy Agents | 20 | Not yet recruiting |

Table 3: Ongoing clinical trials on CAR T-cell therapy in SLE patients
