# Supplementary material for: CAR T cell therapy efficacy and safety in SLE: a systematic review and pooled analysis of 47 patients across 10 studies
Source: Naunyn Schmiedebergs Arch Pharmacol. 2025 Aug 14;399(2):1565–88. doi: 10.1007/s00210-025-04425-z (PMC12901168; doi:10.1007/s00210-025-04425-z)
Supplement: Supplementary file 2 — Supplementary file2 (DOCX 15 KB) [file 210_2025_4425_MOESM2_ESM.docx]

|  | He 2025 | Krickau 2024 | Li 2024 | Hagen 2024 |
| --- | --- | --- | --- | --- |
| Were patient’s demographic characteristics clearly described? | Yes | Yes | Yes | Yes |
| Was the patient’s history clearly described and presented as a timeline? | Yes | Yes | Yes | Yes |
| Was the current clinical condition of the patient on presentation clearly described? | Yes | Yes | Yes | Yes |
| Were diagnostic tests or assessment methods and the results clearly described? | Yes | Yes | Yes | Yes |
| Was the intervention(s) or treatment procedure(s) clearly described? | Yes | Yes | Yes | Yes |
| Was the post-intervention clinical condition clearly described? | Yes | Yes | Yes | Yes |
| Were adverse events or unanticipated events identified and described? | Yes | Yes | Yes | Yes |
| Does the case report provide takeaway lessons? | Yes | Yes | Yes | Yes |

Table 4: Risk of bias assessment of the included case reports
