# Supplementary material for: CAR T cell therapy efficacy and safety in SLE: a systematic review and pooled analysis of 47 patients across 10 studies
Source: Naunyn Schmiedebergs Arch Pharmacol. 2025 Aug 14;399(2):1565–88. doi: 10.1007/s00210-025-04425-z (PMC12901168; doi:10.1007/s00210-025-04425-z)
Supplement: Supplementary file 3 — Supplementary file3 (DOCX 15 KB) [file 210_2025_4425_MOESM3_ESM.docx]

|  | Wang 2024 | Muller 2024 |
| --- | --- | --- |
| Were there clear criteria for inclusion? | Yes | Yes |
| Was the condition measured in a standard, reliable way for all participants? | Yes | Yes |
| Were valid methods used for identification of the condition for all participants? | Yes | Yes |
| Did the case series have consecutive inclusion of participants? | No | Yes |
| Did the case series have complete inclusion of participants? | Unclear | No |
| Was there clear reporting of the demographics of the participants in the study? | Yes | Yes |
| Was there clear reporting of clinical information of the participants? | Yes | Yes |
| Were the outcomes or follow up results of cases clearly reported? | Yes | Yes |
| Was there clear reporting of the presenting site(s)/clinic(s) demographic information? | Yes | Yes |
| Was statistical analysis appropriate? | Yes | Yes |

Table 5: Risk of bias assessment of Wang et al., and Mullar et al,.
